# Sodium dichloroisocyanurate: improving broiler health by reducing harmful microbial levels in the waterline

**DOI:** 10.3389/fvets.2023.1234949

**Published:** 2023-08-01

**Authors:** Qiangqiang Zou, Weishuang Meng, Chunqiang Wang, Tieliang Wang, Xiao Liu, Desheng Li

**Affiliations:** ^1^College of Animal Husbandry and Veterinary Medicine, Jinzhou Medical University, Jinzhou, China; ^2^Liaoning Kaiwei Biotechnology Co., Ltd., Jinzhou, China; ^3^College of Animal Science and Technology, Northeast Agricultural University, Harbin, China

**Keywords:** sodium dichloroisocyanurate, broiler, microorganism, waterline, growth performance

## Abstract

Sodium dichloroisocyanurate (NaDCC) is commonly used for treating drinking water, industrial water, and wastewater. This study aimed to investigate the potential effects of NaDCC-treated waterline drinking water on the growth of AA+ broilers by reducing microbial levels in the waterline. A total of 480 healthy 1-day-old AA+ broilers (46.77 ± 0.50 g) were selected for the experiment and randomly divided into four groups with six replicates of 20 birds each. The control group received regular drinking water, while the test groups received drinking water with NaDCC concentrations of 10, 30, and 50 mg/L. The test groups consumed the treated water on specific days throughout the 42-day experimental period. Results showed that NaDCC treatment significantly reduced the levels of *E. coli*, *Salmonella*, *S. aureus* and *Moulds* in the drinking water at the waterline (*p* < 0.05). Drinking water with NaDCC also led to reduced broiler fecal emissions of NH_3_ and H_2_S, as well as reduced counts of *E. coli*, *Salmonella*, *S. aureus* and *Moulds* (*p* < 0.05), particularly at 30 mg/L and 50 mg/L concentrations. Broilers consuming NaDCC at 50 mg/L exhibited a significant increase in ADG from days 1–42 (*p* < 0.05). The levels of *E. coli*, *Salmonella*, *S. aureus* and *Moulds* in the drinking water at the waterline were significantly and positively correlated with the bacterial count in the feces (*p* < 0.05, R > 0.6). Additionally, bacterial levels in drinking water and broiler feces were negatively correlated with broiler production performance indicators, including ADG, ADFI, F/G and AWC. In conclusion, NaDCC can indirectly enhance broiler performance by reducing the levels of harmful bacteria in the waterline without affecting normal drinking water. The addition of 30 mg/L or 50 mg/L of NaDCC to the waterline in poultry production can effectively control harmful microorganisms and improve poultry health. Based on the experiment’s results, it is recommended to preferentially use 30 mg/L NaDCC in the waterline to reduce farming costs.

## Introduction

Agriculture is an important part of the global economy and livestock production contributes significantly to gross domestic product (1). Poultry meat is becoming one of the most important sources of animal protein for humans in terms of health benefits, cost and production efficiency (2). And pathogenic microorganisms in the water are one of the main culprits of broiler diseases. The use of better water sources and upgraded water treatment processes to enhance drinking water quality has become a widespread practice worldwide, driven by advancements in water purification technology and regulations governing water quality (3). However, despite these efforts, chemical and microbiological contamination of drinking water remains a significant health concern. People strive for pathogen-free drinking water and employ disinfection measures to eliminate most microorganisms from the water. Nonetheless, treated water is not entirely sterile, and small amounts of microorganisms can still be present in drinking water systems (DWS) (4, 5). In the case of livestock, including broilers, the quality of drinking water and DWS play a crucial role in overall health and performance (6, 7).

Research indicates that over 95% of the biomass in water pipes adheres to the pipe walls as biofilm, while only 5% remains suspended in the bulk water (8, 9). Under favorable conditions such as suitable coop temperature, low water flow in the waterline, and sufficient nutrients, DWS can create an ideal environment for microbial growth (10). Common microorganisms found in broiler drinking water (11), including *Campylobacter jejuni*, *E. coli*, *Pseudomonas* and *Salmonella*, have been identified as major biofilm-forming organisms in DWS (12–14). Although biofilms themselves do not contain pathogens, they provide a favorable surface for microbial attachment (15, 16), allowing microorganisms in biofilms to evade clearance mechanisms (17). When pathogen-rich biofilms detach and contaminate the water, they can pose a potential risk to animal and human health if consumed (18).

In daily production practices, various strategies are employed to improve water quality, including acidification and magnetization of drinking water, which have proven effective in inhibiting pathogen growth (19, 20). Treatment with these methods has shown improvements in animal growth performance and reduced pathogen spread (19–21). Chlorinated drinking water is commonly used in the livestock industry due to its ease of application, cost-effectiveness, and broad antimicrobial properties (7). Sodium dichloroisocyanurate (NaDCC), a white powder with a molecular weight of 219.9 g/mol (22), is considered safe and effective for disinfection of drinking water and domestic environments in meat animal treatment (23–25).

NaDCC is widely employed as a bactericide in various setting, such as swimming pool disinfection, drinking water treatment, and industrial deodorization (26, 27). However, there is limited information on the effects of NaDCC waterline disinfection in broiler farming. This experiment hypothesized that the addition of NaDCC to broiler drinking water pipes would enhance broiler health by improving water cleanliness through the reduction of biofilm microbial levels in the pipes. Thus, the primary objective of this study was to evaluate whether the addition of NaDCC to broiler drinking water could enhance broiler health by reducing microbial levels in the waterline. This research will provide a theoretical basis for the future application of NaDCC in broiler production.

## Materials and methods

### Ethics statement

The Animal Conservation and Utilization Committee of the Jinzhou Medical University approved the animal use agreement (Grant No. JZMULL2022011).

### Animals and experimental design

The test waterline had a length of 60 meters, with two test cages at the front, end, and 30 meters in the middle, resulting in a total of six test cages per waterline. Four waterlines comprising a total of 24 test cages, were selected for the experiment. A total of 480 healthy 1-day-old AA+ broilers weighting 46.77 ± 0.50 g were randomly divided into four groups with six replicates of 20 birds per group. The broilers were housed in test cages along each waterline. The experiment lasted for 42 days. In the control group, tap water was provided through the waterline, while in the test groups, the water lines were supplemented with NaDCC at concentrations of 10, 30, and 50 mg/L. The test group consumed tap water containing NaDCC on specific days during the experiment. For the rest of the time, the treatment was the same as the control group. It should be noted that long-term use of chlorinated water can damage the waterline and may have some impact on the broiler organism.

### Material sources

The NaDCC used in this experiment was provided free of charge by Jinzhou Zhongke Genetic Testing Service Co. It was in powder form and had an effective chlorine content of 60.35%.

### Animals feeding management

All birds were housed in experimental cages measuring 1.25 × 0.80 × 0.50 m^3^ per cage, located in a dedicated test broilers room. The test broilers room was fully enclosed and equipped with an automatic environmental control system to maintain optimal temperature and humidity conditions. The temperature was initially set at 33°C and gradually reduced by 3°C every week until reaching 22°C, while the relative humidity was maintained at 65%. The birds had unrestricted access to feed and water. The immunization program for the test broilers include vaccination against Newcastle disease and avian influenza at 7 days of age, followed by a Newcastle disease vaccination at 21 days of age administered through the water. The diets provided to the broiler were formulated to meet the nutritional requirements recommended by the NRC (1994) and were modified to suit the specific nutritional needs of Chinese broilers. The diets were provided in mash form and their composition is detailed in Table 1.

**Table 1:** Composition and nutrient levels of the basal diet.

Items	Contents	
	Days 1–21	Days 22–42
Ingredients (%)^a^		
Corn	60.4	64.05
Soybean meal	34.4	30
CaHPO_4_	1.40	1.30
CaCO_3_	1.21	1.12
NaCl	0.25	0.25
Soybean oil	1.00	2.00
Choline chloride	0.05	0.05
Lysine	0.08	0.10
*DL*-Met	0.21	0.13
Premix	1	1
Total	100.00	100.00
Nutrient levels (%)^b^		
ME (MJ/Kg)	12.14	12.51
CP	21.17	19.24
Available phosphorus	0.38	0.36
Lys	1.29	1.15
Met	0.67	0.48
Met + Cys	1.00	0.72
Ca	0.92	0.87

a Each kg of premix provides: VA, 5000 IU; VD, 10000 IU; VE, 75.0 IU; VK_3_, 18.8 mg; VB_1_, 9.8 mg; VB_2_, 28.8 mg; VB_6_, 19.6 mg; VB_12_, 0.1 mg; Biotin, 2.5 mg; Folic Acid, 4.9 mg; D-Pantothenic acid, 58.8 mg; Nicotinic acid, 196.0 mg; Zn, 37.6 mg; Fe, 40.0 mg; Cu, 4.0 mg; Mn, 50.0 mg; I, 0.2 mg; Se, 0.2 mg.

b The nutrient levels were calculated values.

### Measurement indicators

#### Growth performance

The broilers’ weights were measured at 1 day, 21 days, and 42 days of age. The feed intake was recorded for each replicate throughout the entire experiment, and from this data, average daily feed intake (ADG), average daily weight gain (ADFI), and meat to feed ratio (F/G) were calculated. Additionally, the water consumption of the broilers in each treatment group was carefully recorded, and the average daily water consumption (AWC) was calculated based on this data.

#### Blood indicators

On days 21 and 42 of the experiment, a total of 4 mL of blood was collected from the broiler’s lower wing vein. The blood samples were allowed to stand for 30 min and then centrifuged at 1200 r/min for 15 min to separate the supernatant. The potency of serum antibodies against Newcastle disease and avian influenza H9 was determined using a haemagglutination inhibition test. Furthermore, serum levels of albumin (ALB), total protein (TP), globulin (GLOB), alanine transaminase (ALT), alkaline phosphatase (ALP), and glucose (GLU) were measured using a fully automated biochemical analyzer.

#### Harmful gas emissions

On day 21 and 42 of the experiment, fresh broiler manure samples were collected from each replicate. The emission of ammonia and hydrogen sulphide from the manure were determined using the method described by Dang et al. (28). The collected manure samples were placed in a 2-liter plastic box with small holes attached to the side. The boxes were then left to ferment at room temperature (25°C) for specific durations of 12 h, 24 h, 48 h, and 72 h. Air samples were collected from above the small holes on either side of the box using a gas collection pump. The concentrations of NH_3_ and H_2_S were measured within the range of 0.00–100.00 mg/m^3^.

### Waterline and fecal microbiota

On days 21 and 42 of the experiment, 10 mL of NaDCC-free drinking water was collected from the nipple drinker above each test cage. Additionally, 1 g broiler fecal samples was collected from each replicate and immediately transported to the laboratory on ice, following the method described by Dang et al. (28). The number of *E. coli*, *Salmonella*, *S. aureus* and *Moulds* in the water samples was determined using the plate count method. The number of microorganisms in the waterline was expressed as log_10_ colony forming units per mL of water. Similarly, the number of microorganisms in the fecal samples was expressed as log_10_ colony forming units per gram of feces.

### Data analysis

The data was analyzed using a completely randomized grouping design. Multiple comparisons of significant differences in means were conducted using the one-way ANOVA LSD method in SPSS 25.0, and the results were visualized using Graphpad Prism 8. The results were presented as the mean and standard deviation, with a significance level of *p* < 0.05 indicating a significant difference. Correlation analysis was performed using R (V4.0.2), and the results were displayed using the R (V4.0.2) corrplot package. Pearson correlation analysis was carried out for the 21 and 42-day indicators, and correlations with a significance level of *p* < 0.05 and an absolute correlation coefficient greater than 0.6 or less than-0.6 were considered significant.

## Results

### Growth performance

As shown in Table 2, the addition of 50 mg/L NaDCC to the waterline significantly increased ADG in broilers from 1 to 42 days (*p* < 0.05). The addition of NaDCC at 10 mg/L and 30 mg/L also increased ADG in broilers compared to the control, but not at a significant level (*p* > 0.05). The addition of NaDCC to the waterline had no significant effect on ADFI, F/G, and AWC in broilers (*p* > 0.05). NaDCC supplementation in drinking water had a negative linear effect on F/G in 22–42 day old broilers (*p* = 0.041) and a positive linear effect on ADG in 1–42 day old broilers (*p* = 0.012), with a quadratic curve effect (*p* = 0.027).

**Table 2:** Effect of NaDCC on growth performance of AA+ broiler.

Item		Treatment				*p*-value	
		0 mg/L	10 mg/L	30 mg/L	50 mg/L	Linear	Quadratic
1–21 d	ADG (g/brid)	41.17 ± 1.22	41.46 ± 1.18	41.61 ± 1.19	42.05 ± 1.08	0.184	0.423
	ADFI (g/brid)	51.99 ± 1.68	52.35 ± 1.70	52.56 ± 1.04	53.31 ± 1.33	0.112	0.285
	F/G	1.26 ± 0.01	1.26 ± 0.01	1.26 ± 0.01	1.27 ± 0.03	0.300	0.526
	AWC (l/brid)	0.14 ± 0.02	0.14 ± 0.02	0.14 ± 0.01	0.14 ± 0.01	0.716	0.935
22–42 d	ADG (g/brid)	78.37 ± 2.86	79.86 ± 2.74	81.08 ± 2.63	81.06 ± 2.47	0.078	0.142
	ADFI (g/brid)	133.12 ± 4.12	136.11 ± 4.02	137.11 ± 4.12	136.45 ± 4.36	0.206	0.227
	F/G	1.70 ± 0.01	1.70 ± 0.02	1.69 ± 0.02	1.68 ± 0.01	0.041	0.078
	AWC (l/brid)	0.31 ± 0.03	0.31 ± 0.02	0.30 ± 0.01	0.30 ± 0.01	0.663	0.889
1–42 d	ADG (g/brid)	59.77 ± 1.18^b^	60.66 ± 1.20^ab^	61.34 ± 1.13^ab^	61.56 ± 1.21^a^	0.012	0.027
	ADFI (g/brid)	92.55 ± 1.59	94.23 ± 1.70	94.84 ± 1.69	94.88 ± 2.47	0.054	0.078
	F/G	1.55 ± 0.01	1.55 ± 0.01	1.55 ± 0.01	1.54 ± 0.02	0.129	0.233
	AWC (l/brid)	0.22 ± 0.01	0.22 ± 0.01	0.22 ± 0.01	0.22 ± 0.01	0.812	0.959

^a,b^Means in the different groups with different superscripts are significantly different (*p* < 0.05). Values represent the means of six cages with 1 broiler per replicate cages (*n* = 6) per treatment.

### Blood indicators

**Figure 1:**
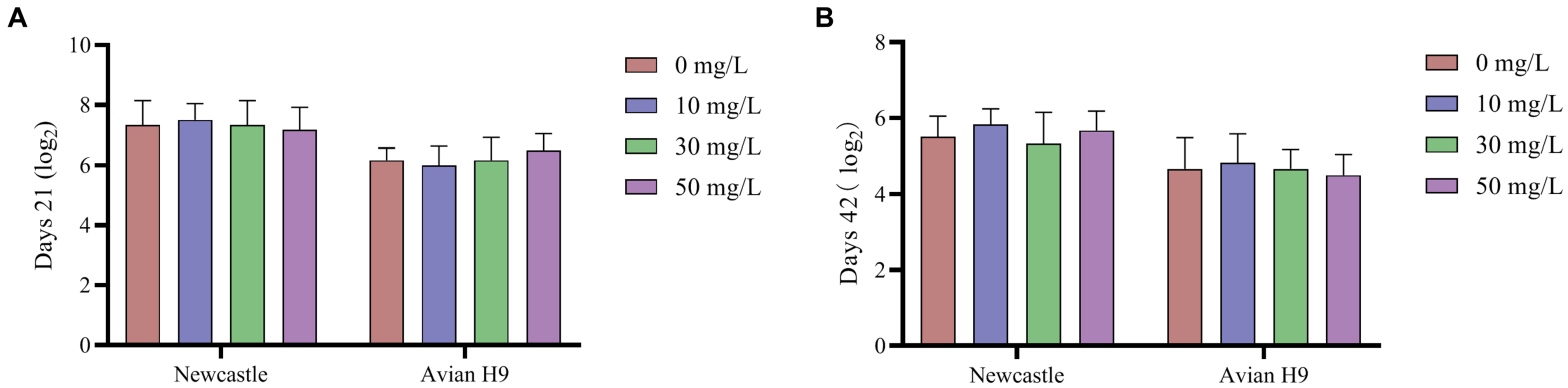
Effect of NaDCC on serum antibody potency of AA+ broiler. **(A)** Days 21 Serum antibody potency; **(B)** Days 42 Serum antibody potency. Values represent the means of six cages with 1 broiler per replicate cages (*n* = 6) per treatment.

**Figure 2:**
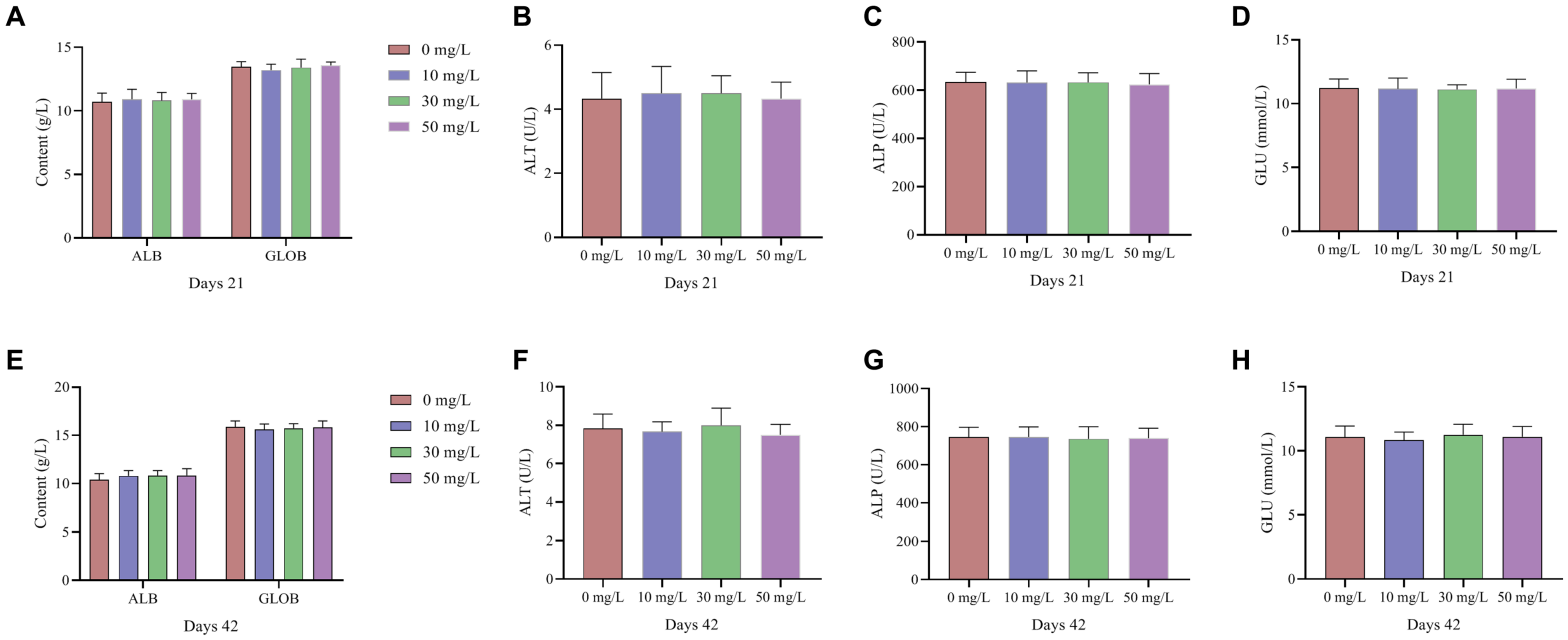
Effect of NaDCC on serum biochemical parameters of AA+ broiler. **(A)** Days 21 Albumin and Globulin. **(B)** Days 21 Alanine transaminase. **(C)** Days 21 Alkaline phosphatase. **(D)** Days 21 Glucose. **(E)** Days 42 Albumin and Globulin. **(F)** Days 42 Alanine transaminase. **(G)** Days 42 Alkaline phosphatase. **(H)** Days 42 Glucose. Values represent the means of six cages with 1 broiler per replicate cages (*n* = 6) per treatment.

As shown in Figures 1, 2, the addition of NaDCC to the waterline did not have a significant effect on antibody potency and various biochemical parameters in broiler serum (*p* > 0.05).

### Harmful gas

**Figure 3:**
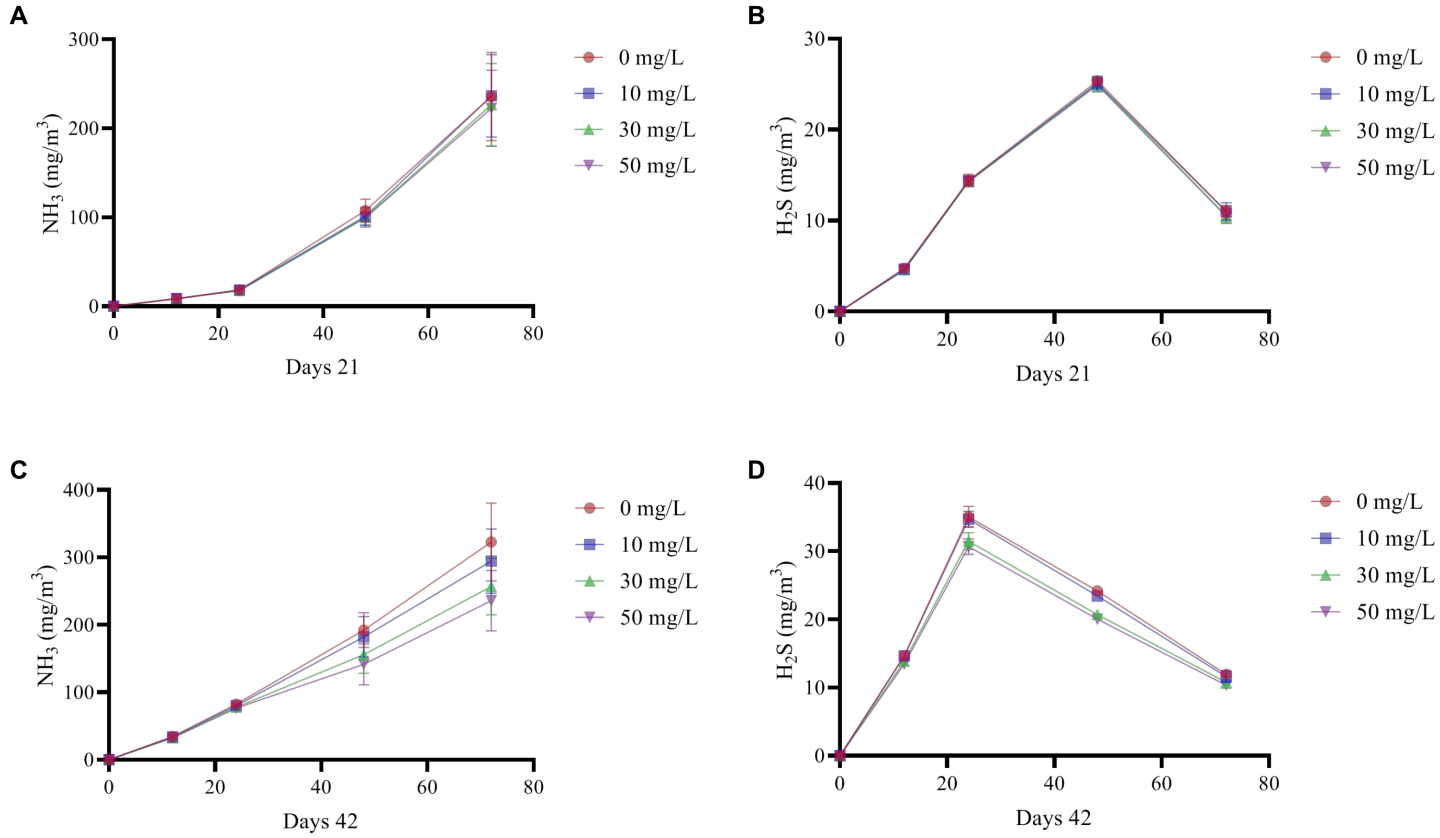
Effect of NaDCC on harmful gas emissions of AA+ broiler. **(A)** Days 21 NH_3_. **(B)** Days 21 H_2_S. **(C)** Days 42 NH_3_. **(D)** Days 42 H_2_S. Values represent the means of six cages with 1 broiler per replicate cages (*n* = 6) per treatment.

As shown in Figure 3, NH_3_ emissions from broiler feces continue to rise over time, while H_2_S emissions increase initially and then decline. Additionally, both NH_3_ and H_2_S emissions were consistently lower in the treatment group compared to the control group.

### Waterline and fecal microbiota

**Figure 4:**
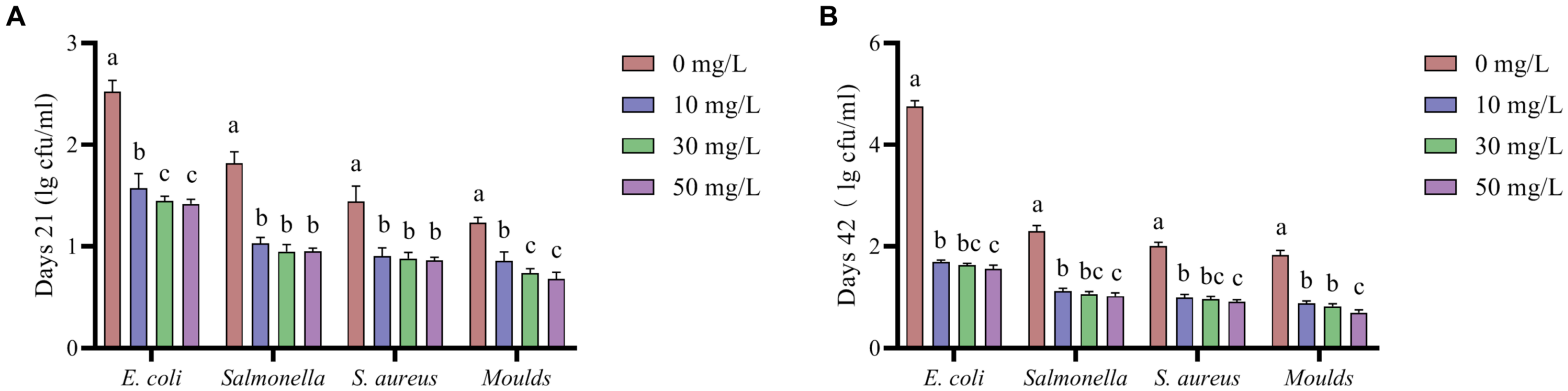
Effect of NaDCC on waterline microbiota of AA+ broiler. **(A)** Days 21 Microbial content. **(B)** Days 42 Microbial content. ^a,b,c^Means in the different groups with different superscripts are significantly different (*p* < 0.05). Values represent the means of six cages with 1 broiler per replicate cages (*n* = 6) per treatment.

According to Figure 4, the addition of NaDCC to the waterline significantly reduced the levels of *E. coli*, *Salmonella*, *S. aureus*, and *Moulds* in the waterline (*p* < 0.05).

**Figure 5:**
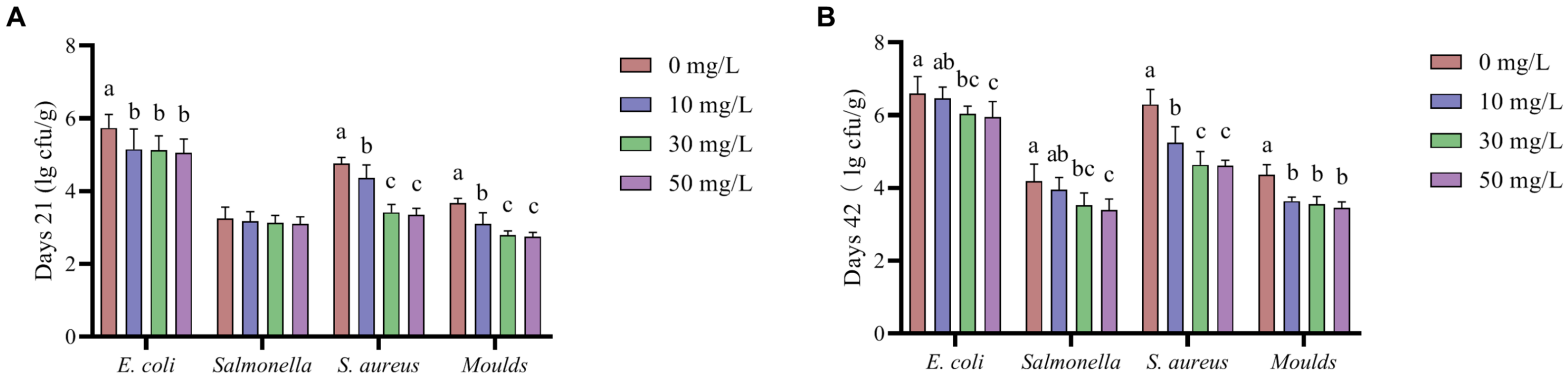
The effect of NaDCC on the microbial content of AA+ broiler feces. **(A)** Days 21 Microbial content. **(B)** Days 42 Microbial content. ^a,b,c^Means in the different groups with different superscripts are significantly different (*p* < 0.05). Values represent the means of six cages with 1 broiler per replicate cages (*n* = 6) per treatment.

As shown in Figure 5, the addition of NaDCC to the waterline resulted in significantly lower levels of *E. coli*, *S. aureus* and *Moulds* in broiler feces at 21 and 42 days (*p* < 0.05). The addition of NaDCC to the waterline reduced *Salmonella* levels in broiler feces at 21 days, but not to a significant level (*p* > 0.05), while it significantly reduced *Salmonella* levels in broiler feces at 42 days (*p* < 0.05).

### Correlation analysis of microorganisms and production performance

**Figure 6:**
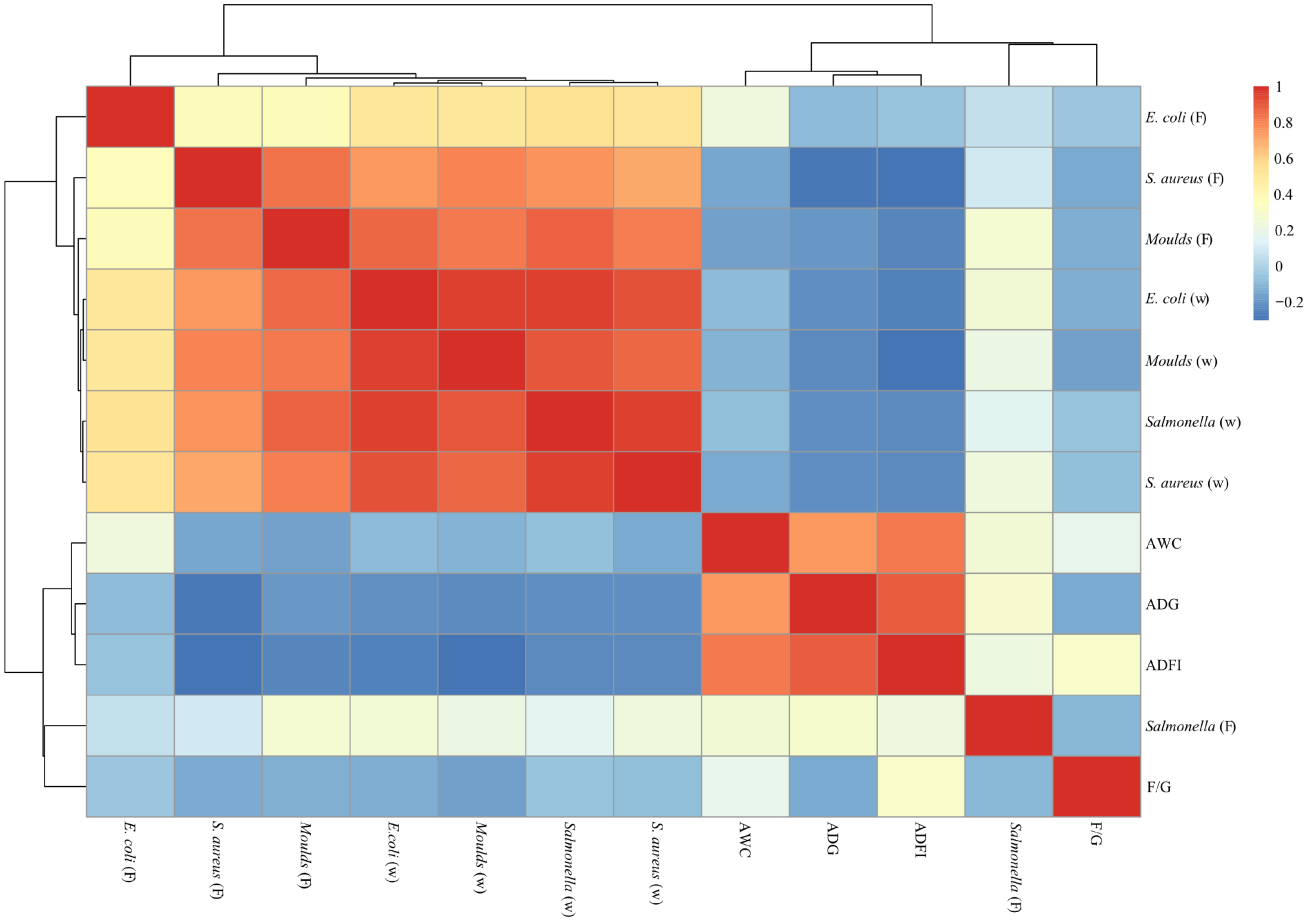
Correlation analysis of microorganisms and production performance on days 21. “W” means microbial content in the waterline; “F” means microbial content in the feces. The same as below.

**Figure 7:**
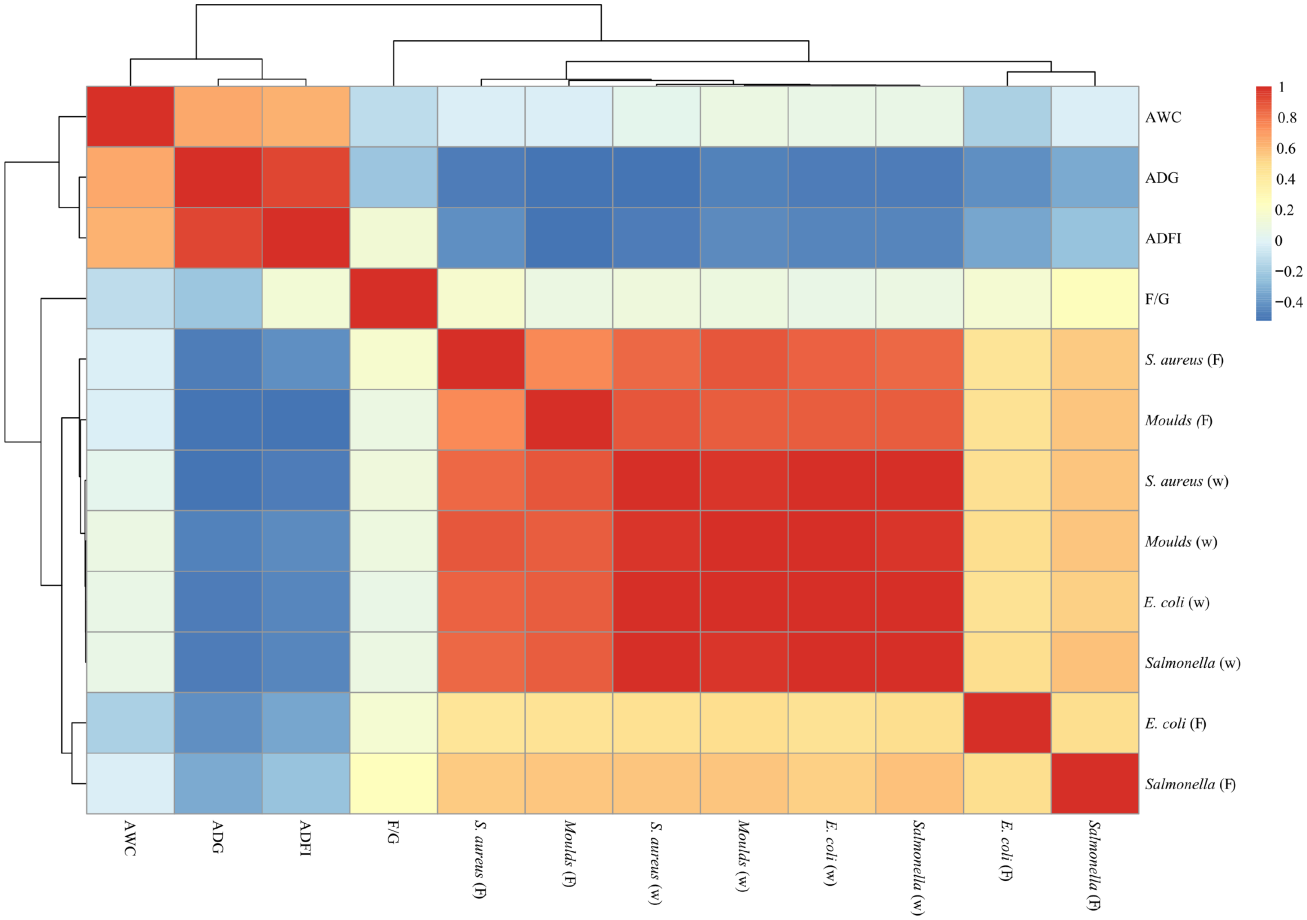
Correlation analysis of microorganisms and production performance on days 42.

As shown in Figures 6, 7, a significant positive correlation (*p* < 0.05, R > 0.6) was observed between the microbial content (*E. coli*, *Salmonella*, *S. aureus* and *Moulds*) in the waterline and feces. However, the microbial content in the waterline and manure showed a negative correlation with production performance indicators (ADG, ADFI, F/G and AWC), although these correlations did not reach significant levels (*p* > 0.05).

## Discussion

### Effect of sodium dichloroisocyanurate on microorganisms in waterline and broiler feces

Drinking water and drinking water systems play an important role in the overall health and performance of broiler chickens (29). This pilot study found that the addition of NaDCC to broiler drinking water systems significantly reduced the levels of *E. coli*, *Salmonella*, *S. aureus*, and *Moulds* in both the waterline and broiler feces. Correlation analysis revealed a significant positive correlation between microbial levels in the waterline and feces. Generally, bacterial infections in broilers are caused by microorganisms commonly found in the poultry environment. In this context, colibacillosis caused by *E. coli* (30), paratyphoid infection caused by *Salmonella* (31), necrotizing enteritis caused by *Clostridium perfringens* (32), as well as *S. aureus* infection (33) and *Pseudomonas aeruginosa* infection (34) could be important contaminants leading to issues in 3–6 week old chickens.

NaDCC is a highly effective disinfectant containing two active chlorine atoms that disrupt cell membranes, nucleic acids, and proteins, thereby causing oxidative degradation of microorganisms (35). It has been reported to immediately kill *E. coli*, *S. aureus*, and *fungi* (36). Jang et al. (37) demonstrated that a 0.3% NaDCC solution effectively kills *Salmonella* even at −10°C after only 1 min of treatment, while a 0.1% glutaraldehyde solution requires 10 min of treatment at 25°C. Ferreira et al. (38) found that using a disinfectant containing 0.67 g/L sodium dichloroisocyanurate for 15 min effectively kills *Pseudomonas aeruginosa*, *E. coli*, *Salmonella*, and *Clostridium perfringens* in disinfection tests. Increasing the concentration to 0.93 g/L and treating for 20 min eliminated all *Staphylococcus aureus*. In this experiment, the addition of NaDCC to the drinking water of broilers demonstrated a reduction in the number of pathogenic bacteria entering the broiler by reducing the amount of these bacteria in the waterline, consequently leading to a decrease in pathogenic bacteria in the feces.

### Effect of sodium dichloroisocyanurate on the health of broiler organisms

Drinking clean and hygienic water is essential for the welfare of broilers. This pilot study found that drinking water containing NaDCC did not affect the normal water intake of broilers. However, it significantly increased the ADG when consumed by broilers at a concentration of 50 mg/L. Nonetheless, at this stage, there is insufficient information to confirm that NaDCC can directly increase the body weight of broilers. Based on the results of this experiment, it is hypothesized that the potential cause of weight gain in broilers is the addition of NaDCC to their drinking water, which eliminates pathogenic microorganisms in the drinking water and waterline biofilm. When chickens consume healthy and clean water, it reduces their intake of pathogenic bacteria, leading to an increase in beneficial bacteria in their gut. By improving the micro-ecological environment of the gut, broilers tend to have increased feed intake and subsequently gain more body weight. However, the exact mechanism underlying this hypothesis requires further in-depth study.

It has been demonstrated that birds can tolerate free chlorine residues of 50 ppm in drinking water without any adverse effects (39, 40). Similar results were obtained in this experiment, as the broiler serum antibodies and biochemical parameters were not affected by the drinking water containing NaDCC. More-Bayona et al. (41) reported an increase in macrophages and a decrease in CD8+ lymphocytes in the peritoneal cavity of broilers that consumed well water containing a mixture of biological preparations for an extended period. They also observed varying degrees of inflammatory responses in the organism caused by fungi and bacteria present in the water. Therefore, occasional consumption of chlorinated drinking water is necessary for the health of broilers.

Under anaerobic conditions, microbial decomposition of feces produces certain gaseous compounds, mainly from carbohydrates and proteins (42). In this experiment, drinking water containing NaDCC significantly reduced NH_3_ and H_2_S emissions from feces. Previous reports have shown that when the number of *E. coli* and *Salmonella* in feces is reduced, fecal NH_3_ and H_2_S emissions are also reduced (43). Prolonged exposure of broilers to NH_3_ has been shown to affect the structure of their cecal flora and, consequently, their growth performance (44). Additionally, sudden releases of H_2_S from manure stored in chicken houses can increase the concentration of toxic substances in the room, leading to the death of many animals and humans (45). While birds are not as sensitive to the toxic effects of hydrogen sulfide as mammals, high concentrations of this gas in the air can still negatively impact production parameters in laying hens and broilers (46). Therefore, improving the farm environment is equally important for the healthy growth of broilers. Based on the results of this experiment, NaDCC is highly effective in waterline treatment. Further research can focus on optimizing the dosage to reduce resource consumption while maintaining efficacy.

## Conclusion

The addition of sodium dichloroisocyanurate to broiler drinking water systems does not impact broiler feed intake, immune function, or the normal physiological functions of the organism. By reducing the levels of pathogenic microorganisms in the waterline, it is possible to also reduce their presence in broiler feces, ultimately improving the overall growth of the broilers. Taking all factors into consideration, it is recommended to use a concentration of 30 mg/L of sodium dichloroisocyanurate in poultry production to enhance the health of broilers.

## Data availability statement

The raw data supporting the conclusions of this article will be made available by the authors, without undue reservation.

## Ethics statement

The animal study was reviewed and approved by Animal Ethics Committee of Jinzhou Medical University.

## Author contributions

QZ: data curation and writing-original draft. WM: investigation. CW: writing-review and editing. TW: resources. XL: project administration, supervision, and validation. DL: resources, supervision, validation, writing, and writing-review and editing. All authors contributed to the article and approved the submitted version.

## Funding

This work was funded by the 2022 Proposed Student Innovation and Entrepreneurship Project (Grant no. S202210160027).

## Conflict of interest

TW was employed by Liaoning Kaiwei Biotechnology Co., Ltd.

The remaining authors declare that the research was conducted in the absence of any commercial or financial relationships that could be construed as a potential conflict of interest.

## Publisher’s note

All claims expressed in this article are solely those of the authors and do not necessarily represent those of their affiliated organizations, or those of the publisher, the editors and the reviewers. Any product that may be evaluated in this article, or claim that may be made by its manufacturer, is not guaranteed or endorsed by the publisher.
